# Efficacy and Safety of Intranasal Etripamil for Paroxysmal Supraventricular Tachycardia: Meta-Analysis of Randomized Controlled Trials

**DOI:** 10.3390/jcm14113720

**Published:** 2025-05-26

**Authors:** Mayank Jha, David Song, Andrew Kung, Sam Lo, Alexander Sacher, Song P. Ang, Aasim Akthar, Hritvik Jain, Raheel Ahmed, Matthew Bates, Sang Lee, Seth Goldbarg

**Affiliations:** 1 Department of Internal Medicine, Government Medical College and New Civil Hospital, Surat 395001, India; 2 Division of Cardiology, New York Presbyterian Queens, Flushing, NY 11355, USA; 3 Division of Internal Medicine, New York Presbyterian Queens, Flushing, NY 11355, USA; 4 Department of Internal Medicine, Icahn School of Medicine, Mt. Sinai Elmhurst Hospital Center, New York, NY 10029, USA; 5 Department of Internal Medicine, Rutgers Health/Community Medical Center, Toms River, NJ 08755, USA; 6 Department of Internal Medicine, St. Francis Medical Center, Monroe, LA 71201, USA; 7 Department of Internal Medicine, All Indian Institute of Medical Sciences (AIIMS), New Delhi 110029, India; 8 National Heart and Lung Institute, Imperial College London, London SW3 6LY, UK; 9 Department of Electrophysiology, James Cook University Hospital, Middlesbrough TS4 3BW, UK; 10 Director of Cardiac Electrophysiology, New York Presbyterian Queens, Flushing, NY 11355, USA

**Keywords:** efficacy, intranasal etripamil, cardiac arrhythmias, paroxysmal supraventricular tachycardia, meta-analysis, adverse drug events

## Abstract

**Background:** Patients with arrhythmias, particularly paroxysmal supraventricular tachycardia (PSVT), face an increased risk of cardiac complications. Currently, non-parenteral medications for rapid PSVT cessation are lacking. Etripamil, a novel intranasal, short-acting calcium channel blocker, offers a rapid onset and the potential for unsupervised PSVT management. However, data on its use in arrhythmia management remain limited. **Aims:** We aimed to assess the efficacy and safety of 70 mg of etripamil compared with placebo in the treatment of PSVT. **Methods:** We systematically searched PubMed, Embase, Web of Science, Scopus, and the Cochrane Library for randomized controlled trials (RCTs) from inception to April 2025. We calculated pooled risk ratios (RRs) with 95% confidence intervals (CIs) using random or common effects models, depending on the heterogeneity. **Results:** Four RCTs including 540 patients were analyzed. Etripamil demonstrated higher conversion rates to the sinus rhythm at 15 min (RR 1.84 [95% CI: 1.32–2.48]), 30 min (RR 1.80 [95% CI: 1.38–2.35]), and 60 min (RR 1.24 [95% CI: 1.04–1.48]). PSVT recurrence rates were similar between groups (RR 0.52 [95% CI: 0.20–1.34]). Adverse events (AEs) and severe AEs were comparable between etripamil and the placebo. Etripamil was associated with higher rates of nasal discomfort, nasal congestion, rhinorrhea, and epistaxis but not with increased bradyarrhythmia, atrial fibrillation, or non-sustained ventricular tachycardia. **Conclusions:** Etripamil appears to be a promising treatment for cardiac arrhythmias. Larger long-term RCTs are needed to confirm its safety and efficacy in clinical practice.

## 1. Introduction

Paroxysmal supraventricular tachycardia (PSVT) is defined as a regular, typically narrow-complex tachyarrhythmia that arises from or conducts through the atria or the atrioventricular node and is commonly encountered in clinical practice. PSVT often results from a reentry circuit and can be categorized by its anatomic location, with two common types: atrioventricular nodal reentrant tachycardia (AVNRT) and atrioventricular reentrant tachycardia (AVRT) [1]. AVNRT, the most common subtype, occurs when electrical impulses travel through two pathways within the AV node, while AVRT involves reentry via an accessory pathway. Although PSVT is usually not life-threatening, its sudden, disruptive, and sometimes disabling symptoms can significantly impair one’s quality of life [2].

Based on retrospective analyses of American clinical databases, it is estimated that PSVT affects approximately 1 in 300 individuals, corresponding to about 1.26 million people in the United States, with the highest rates observed among older and female patients [1,2]. Patients often present with abrupt-onset palpitations, chest discomfort, dizziness, or dyspnea. Diagnosis is aided by 12-lead electrocardiograms (EKGs) obtained during arrhythmic events or a sinus rhythm to identify arrhythmogenicity and guide further management, in line with ACC, AHA, and HRS guidelines [3].

Current guidelines recommend first-line treatment of PSVT in an acute setting with vagal maneuvers or adenosine, followed by beta blockers or non-dihydropyridine calcium channel blockers (CCBs), such as verapamil or diltiazem, for stable PSVT and synchronized cardioversion for unstable PSVT [4]. However, these therapies require hospitalization, highlighting the need for a safe, rapid, and effective treatment option suitable for outpatient use. The intranasal route offers a compelling alternative, as the posterior nasal mucosa enables rapid systemic drug absorption [5]. In terms of interventional management, percutaneous catheter ablation is recommended as first-line therapy for PSVT in international guidelines [6,7]. Although ablation achieves high success rates (97% in AVNRT and 93% in AVRT), it carries procedural risks and may not be accessible or acceptable to all patients [8]. Available oral therapies, meanwhile, carry risks of hypotension and heart failure exacerbation, highlighting the need for novel therapeutic options [5].

Recent research has focused on developing a nonparenteral medication capable of rapid PSVT termination and suitable for patient self-administration outside healthcare settings. Etripamil, an intranasal, non-dihydropyridine L-type CCB, has a rapid onset of action, short half-life, and minimal systemic adverse effects compared with intravenous agents [9]. Clinical trials have suggested that intranasal etripamil could provide a safe, practical outpatient option for acute self-termination of paroxysmal SVT without the severe systemic adverse effects that come with intravenous drugs [10,11].

The NODE-301 trial, a phase 3, multicenter, double-blind, placebo-controlled study, demonstrated that etripamil was superior to a placebo in converting PSVT to a sinus rhythm [12]. The subsequent study conducted across North America and Europe included 184 patients who tolerated a test dose of 70 mg of intranasal etripamil [13]. Participants self-administered an initial dose of etripamil or a placebo and a second dose if symptoms persisted. Etripamil also reported higher rates of conversion back to the sinus rhythm and a shorter time to conversion [14]. Etripamil was generally well tolerated, with adverse effects limited to mild, transient nasal symptoms.

Given these promising results, we conducted a meta-analysis of randomized controlled trials to evaluate the efficacy and safety of intranasal etripamil for the treatment of cardiac arrhythmias.

## 2. Methodology

We conducted a systematic review and meta-analysis while following the Preferred Reporting Items for Systematic Reviews and Meta-Analyses (PRISMA) guidelines (Supplementary Tables S1 and S2) and the *Cochrane Handbook of Systematic Reviews and Meta-Analysis* [15,16]. The prespecified study protocol has been registered with PROSPERO (CRD42024544315).

### 2.1. Search Strategy

We systematically searched PubMed, EMBASE, Web of Science, SCOPUS, and the Cochrane Library for relevant articles from inception until April 2025. The search targeted studies evaluating the efficacy of intranasal etripamil for ventricular arrhythmias. No restrictions were applied regarding language or publication year. We also searched the gray literature and reviewed the reference lists of relevant studies. When necessary, the study authors were contacted for additional data.

We performed medical subject heading (MeSH) and keyword searches using Boolean operators (AND, OR). The search strategy combined the following terms: ((etripamil) AND (“supraventricular tachycardia” OR SVT OR “paroxysmal supraventricular tachycardia” OR PSVT OR “cardiac arrhythmia”)). All references were downloaded for consolidation, duplicate removal, and further analysis.

### 2.2. Eligibility Criteria

Eligible studies met the following criteria:

1. Randomized controlled trials (RCTs);
2. Inclusion of patients with PSVT;
3. Administration of etripamil (70 mg) via the intranasal route;
4. English language publication’
5. Reporting of primary clinical outcomes, including conversion to a sinus rhythm or recurrence of arrhythmic events.

We excluded studies which met the following criteria:

1. Did not include adult human participants;
2. Lacked safety data on adverse events (AEs), treatment-emergent serious adverse events (TESAEs), or other prespecified outcomes (nasal discomfort, epistaxis, etc.);
3. Did not have a comparator group;
4. Did not report the timepoints for conversion to a normal sinus rhythm;
5. Had incomplete or unpublished data, abstracts without full peer-reviewed publications, or registered protocols without results.

Two investigators (M.J. and D.S.) independently screened titles and abstracts for relevance. Full-text articles were assessed against the inclusion and exclusion criteria. Discrepancies were resolved through discussion or adjudication by a third reviewer (S.P.). Study selection was documented using a PRISMA flow diagram.

### 2.3. Data Extraction

Two reviewers independently extracted data into a standardized Excel spreadsheet. The extracted variables included the first author, publication year, country, number of participants, and baseline characteristics (age, sex, and body mass index [BMI]). The outcomes collected were the rates of conversion to a sinus rhythm at 15, 30, 60, and 300 min post administration; recurrence of PSVT; and incidence of AF, bradyarrhythmia, and NSVT. The rates of conversion were study design-based and used in the studies included in our meta-analysis. We also extracted the AE, TESAE, nasal discomfort, nasal congestion, epistaxis, and rhinorrhea rates. Any discrepancies were resolved through discussion.

### 2.4. Risk of Bias Assessment

We assessed the risk of bias using the Revised Cochrane Collaboration’s Risk of Bias (RoB-2) tool v0.3.0 [17]. This tool evaluates bias related to randomization, deviations from intended interventions, missing outcome data, outcome measurement, and selective reporting. We visualized the risk of bias assessments using the ROBVIS tool [18]. Given the limited number of included studies (*n* = 4), we did not assess publication bias.

### 2.5. Statistical Analysis

We conducted meta-analyses using RevMan software version 5.4.1. We pooled dichotomous outcomes and calculated risk ratios (RRs) with corresponding 95% confidence intervals (CIs) as the effect measures. Analyses were initially performed using fixed effect models. Random effect models were applied when significant heterogeneity was detected. We assessed heterogeneity using the chi-square test (with a significance level of α < 0.1) and quantified it using Higgins’ I^2^ statistic, with I^2^ > 50% considered indicative of substantial heterogeneity [19]. In cases of significant heterogeneity, we performed sensitivity analyses by sequentially excluding individual studies to explore potential sources.

## 3. Results

### 3.1. Study Selection

Our search identified 72 records (PubMed: 20; Scopus: 29; Cochrane Library: 23). After removing 28 duplicates, we screened the titles and abstracts, excluding those that did not meet the eligibility criteria. Thirty full-text articles were assessed, and 12 were excluded for reasons detailed in the PRISMA flowchart (Supplementary Figure S1). Ultimately, four studies met the inclusion criteria and were included in this systematic review [20,21,22,23].

### 3.2. Study and Patient Characteristics

The study characteristics and baseline patient demographics are shown in Table 1. The included trials were conducted in Belgium, Canada, Hungary, the Netherlands, and the United States, with publication dates ranging from 2018 to 2023. Together, the four trials enrolled 540 patients, of whom 199 were male (36.9%). The largest study included 196 participants (35.9%), and the smallest one had 56 participants (10.4%). The mean age of the participants ranged from 52.2 to 64.6 years. Two trials reported body mass index (BMI) data, with the mean BMI ranging between 29.2 and 29.4 kg/m^2^.

### 3.3. Primary Clinical Outcomes

**Figure 1:**
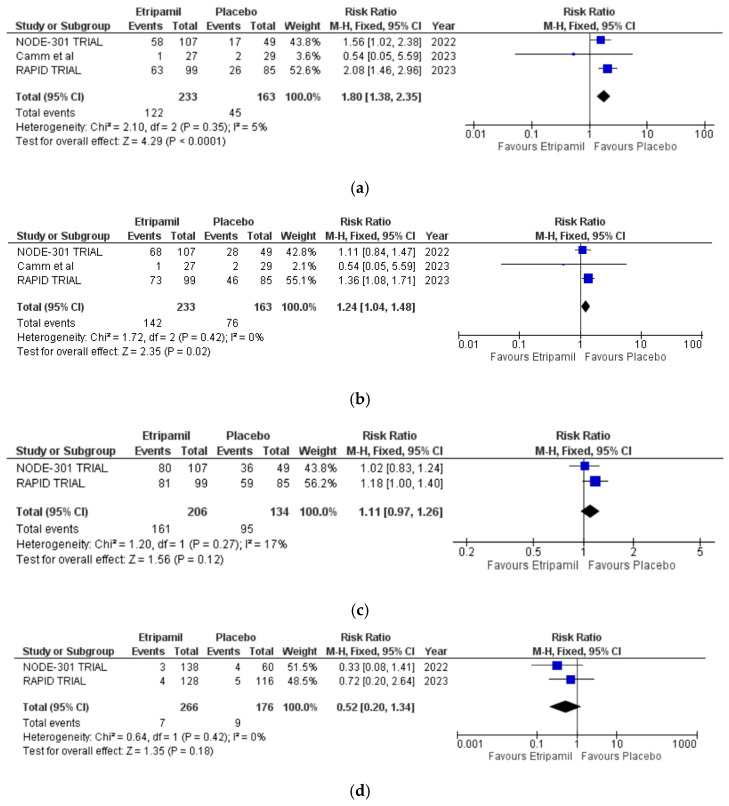
Forest plots for primary clinical outcomes demonstrating the conversion to a sinus rhythm [23]. (**a**) The total number of participants converted at 30 min. (**b**) The total number of participants converted at 60 min. (**c**) The total number of participants converted at 300 min. (**d**) Recurrence of PSVT.

Three trials reported rates of conversion to a sinus rhythm at 15, 30, and 60 min post administration. At 15 min, 106 of the 229 patients (46.3%) in the etripamil group converted to a sinus rhythm, compared with 40 of the 154 patients (26.0%) in the placebo group. Etripamil was associated with a higher rate of conversion to a sinus rhythm at 15 min (RR = 1.84 [95% CI: 1.32–2.48], I^2^ = 0%). At 30 min, 122 of 233 patients (52.3%) in the etripamil group converted to sinus rhythm, compared with 45 of 163 patients (27.7%) in the placebo group. Etripamil was associated with a higher rate of conversion to a sinus rhythm at 30 min (RR = 1.80 [95% CI: 1.38–2.35], I^2^ = 5%) (Figure 1a).

At 60 min, 142 of the 233 patients (60.9%) in the etripamil group achieved conversion to a sinus rhythm, compared with 76 of 163 patients (46.6%) in the placebo group. Etripamil was associated with a higher rate of conversion to a sinus rhythm at 60 min (RR = 1.24 [95% CI: 1.04–1.48], I^2^ = 0%) (Figure 1b).

Two trials reported conversion to a sinus rhythm at 300 min. At 300 min, 161 of the 206 patients (78.2%) in the etripamil group achieved conversion to a sinus rhythm, compared with 95 of 134 patients (70.9%) in the placebo group. Etripamil was not associated with a significantly higher rate of conversion to a sinus rhythm at 300 min (RR = 1.11 [95% CI: 0.97–1.26], I^2^ = 17%) (Figure 1c).

Two studies reported recurrence rates of PSVT after the administration of etripamil or placebo. The recurrence rate was 7 of the 266 patients (2.6%) in the etripamil group, compared with 9 of 175 patients (5.1%) in the placebo group. Etripamil was not associated with an increased rate of PSVT recurrence (RR = 0.52 [95% CI: 0.20–1.34], I^2^ = 0%) (Figure 1d).

**Figure 2:**
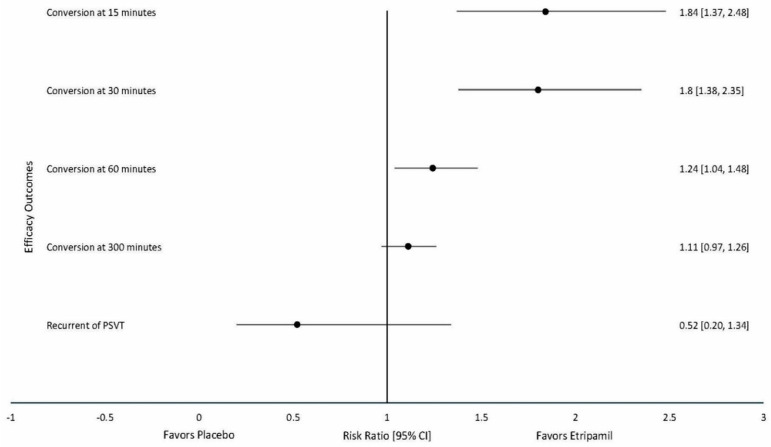
Summary of estimates of meta-analysis of clinical trials comparing intranasal etripamil for the prevention of cardiac arrhythmias.

The pooled risk ratio for the combined effect is depicted in Figure 2, which generally favors etripamil.

### 3.4. Secondary Outcomes

**Figure 3:**
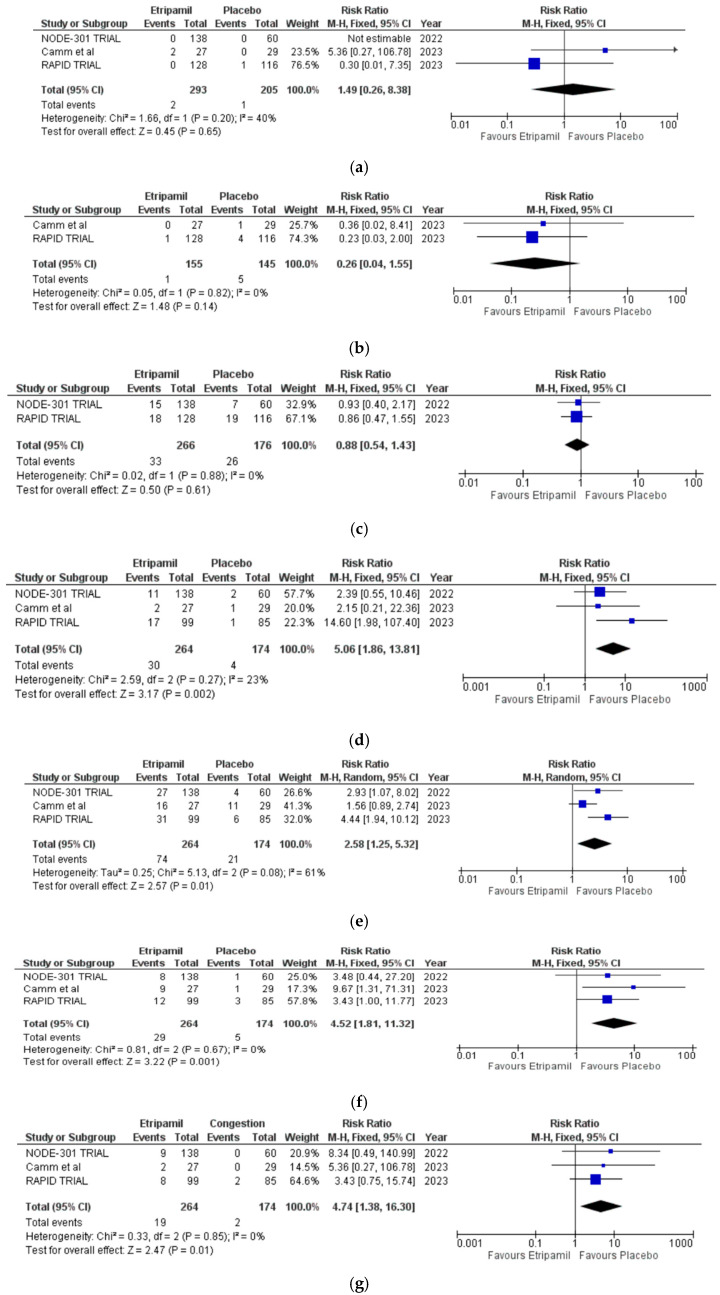

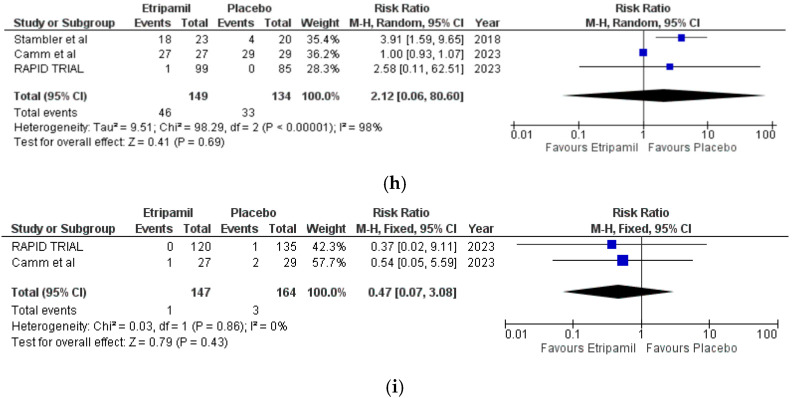
Forest plots demonstrating the secondary safety outcomes for intranasal etripamil [20,23]. (**a**) bradyarrhythmia, (**b**) atrial fibrillation, (**c**) non-sustained ventricular tachycardia, (**d**) nasal congestion, (**e**) nasal discomfort, (**f**) rhinorrhea, (**g**) epistaxis, (**h**) AAEs, and (**i**) TESAEs.

Two studies reported rates of bradyarrhythmia, atrial fibrillation (AF), and non-sustained ventricular tachycardia (NSVT). In the etripamil group, 2 of 155 patients (1.3%) had bradyarrhythmia, 1 of 155 (0.6%) had AF, and 33 of 266 (12.4%) had NSVT. In the placebo group, 1 of 145 patients (0.7%) had bradyarrhythmia, 5 of 145 (3.4%) had AF, and 26 of 176 (14.8%) had NSVT. Etripamil was not associated with an increased incidence of bradyarrhythmia (RR = 1.49 [95% CI: 0.26–8.38], I^2^ = 40%), AF (RR = 0.26 [95% CI: 0.04–1.55], I^2^ = 0%), or NSVT (RR = 0.88 [95% CI: 0.54–1.43], I^2^ = 0%) (Figure 3a–c).

Three studies reported the incidence of nasal discomfort, nasal congestion, rhinorrhea, and epistaxis. In the etripamil group, 74 of 264 patients (28.0%) reported nasal discomfort, 30 of 264 (11.4%) reported nasal congestion, 29 of 264 (11.0%) reported rhinorrhea, and 19 of 264 (7.2%) reported epistaxis. In the placebo group, 21 of 174 patients (12.1%) reported nasal discomfort, 4 of 174 (2.3%) reported nasal congestion, 5 of 174 (2.9%) reported rhinorrhea, and 2 of 174 (1.1%) reported epistaxis. Etripamil was associated with a higher incidence of nasal congestion (RR = 5.06 [95% CI: 1.86–13.81], I^2^ = 23%), nasal discomfort (RR = 2.58 [95% CI: 1.25–5.32], I^2^ = 61%), rhinorrhea (RR = 4.52 [95% CI: 1.81–11.32], I^2^ = 0%), and epistaxis (RR = 4.74 [95% CI: 1.38–16.30], I^2^ = 0%) (Figure 3d–g).

Three trials reported the rates of any adverse events (AEs) and treatment-emergent serious adverse events (TESAEs). In the etripamil group, 46 of 149 patients (30.9%) reported AEs, compared with 33 of 134 (24.6%) in the placebo group. Etripamil was not associated with a higher incidence of AEs (RR = 2.12 [95% CI: 0.06–80.60], I^2^ = 98%) (Figure 3g). Sensitivity analysis following the removal of the study by Camm et al. [23] showed that the heterogeneity dropped substantially from 98% to 0%. However, the risk ratio increased to 3.79 [95% CI: 1.59–9.04] [23] (Supplementary Figure S2).

TESAEs occurred in 1 of 147 patients (0.7%) in the etripamil group, compared with 3 of 164 (1.8%) in the placebo group. Etripamil was not associated with a higher rate of TESAEs (RR = 0.47 [95% CI: 0.07–3.08], I^2^ = 0%) (Figure 3h).

### 3.5. Risk of Assessment

All four RCTs [20,21,22,23] reported the primary clinical outcome of conversion to a sinus rhythm at 15, 30, 60, or 300 min following intranasal etripamil administration. The Revised Cochrane Collaboration’s Risk of Bias (RoB-2) tool was used to evaluate the risk of bias [18]. Risk of bias assessments indicated low risk or minor concerns across the studies (Supplementary Figure S3). Each trial employed centralized randomization procedures with appropriate allocation concealment, minimizing the risk of selection bias. Blinding of the participants was adequately performed using matched placebos, thereby reducing the potential for bias. Outcome data were nearly complete in all studies. Additionally, all reported outcomes corresponded with prespecified protocols, limiting the risk of selective reporting.

## 4. Discussion

The objective of this study was to assess the efficacy and safety of 70 mg of etripamil compared with placebo in the treatment of PSVT. The incidence of PSVT is approximately 36 per 100,000 persons and is notably female predominant. Vagal maneuvers are currently recommended as a first-line treatment for SVT. However, their efficacy is highly variable [24]. When vagal maneuvers fail, pharmacological therapies such as adenosine, intravenous non-dihydropyridine calcium channel blockers, or beta blockers are used for stable SVT [25], typically requiring in-patient management. Our pooled analysis revealed that etripamil significantly improved conversion to a sinus rhythm at 15, 30, and 60 min without an associated increase in adverse events (AEs) or treatment-emergent serious adverse events (TESAEs). PSVT recurrence rates and overall adverse events, including severe events, were similar between groups. However, etripamil was linked to more nasal side effects (discomfort, congestion, rhinorrhea, and epistaxis) without an increased risk of serious cardiac arrhythmias. Etripamil appears to be a relatively safer and more effective alternative to current systemic treatments, especially given its potential for outpatient use. Unlike systemic interventions that necessitate inpatient monitoring, etripamil has demonstrated superior efficacy and safety when administered intranasally in an outpatient setting.

Among key clinical trials, the NODE-301 study showed a conversion rate of 53.7% to a sinus rhythm following a single intranasal dose of etripamil. However, it did not demonstrate a significant benefit at 300 min post administration. Consistently, our pooled analysis revealed that at the 5 h mark, the arrhythmia converted into a sinus rhythm, but this was not statically significant. The RAPID trial, a sequel to NODE-301, investigated a double-dose strategy with two administrations of intranasal etripamil 10 min apart, leading to improved conversion rates at both 30 min (HR = 2.62 [95% CI: 1.66–4.15]) and 300 min (HR = 1.70 [95% CI: 1.21–2.38]). Nevertheless, when these data were pooled in our analysis, the conversion rate at 300 min did not reach statistical significance (RR = 1.11 [95% CI: 0.97–1.26]).

Our updated meta-analysis, including over 540 participants and incorporating studies up to April 2025, builds upon prior systematic reviews such as that by Abuelazm et al. [26]. Consistent with their findings, etripamil demonstrated a statistically significant conversion to a sinus rhythm at 15, 30, and 60 min. While both analyses observed a higher incidence of AEs with etripamil use, the difference was not statistically significant in our results (30.9% in the etripamil group versus 24.6% in the placebo group; RR = 2.12 [95% CI: 1.38–2.35]) [26].

Current 2020 AHA guidelines recommend adenosine as a first-line agent for PSVT. Although adenosine effectively terminates SVT by transiently blocking conduction through the AV node, it is associated with significant side effects such as bronchospasm, facial flushing, chest discomfort, and a sense of impending doom [25]. Comparative analyses, including the systematic review by Delaney et al., have shown that adenosine has a higher rate of adverse events compared with verapamil, which in turn has a higher incidence of hypotension [27]. Compared with these systemic therapies, etripamil offers the advantages of rapid onset, intranasal delivery, and the potential for self-administration without medical supervision.

The REVERT trial demonstrated improved efficacy in SVT termination, using a modified Valsalva maneuver compared with the standard maneuver. However, the modified Valsalva maneuver requires healthcare provider assistance, whereas etripamil can be self-administered [28]. Although the modified Valsalva maneuver is effective, prior reviews suggested that etripamil provides superior success rates in PSVT termination [24,25,26].

Etripamil’s adverse events were primarily local and self-limited, with higher rates of nasal discomfort, nasal congestion, rhinorrhea, and epistaxis compared with placebo. Nevertheless, episodes of nasal side effects were transient, and no serious long-term complications were reported. However, none of our included studies discussed whether the adverse events were self-limiting or contributed to patient compliance. Importantly, etripamil was not associated with an increased incidence of bradyarrhythmias (RR = 1.49 [95% CI: 0.26–8.38]), atrial fibrillation (RR = 0.26 [95% CI: 0.04–1.55]), or non-sustained ventricular tachycardia (RR = 0.88 [95% CI: 0.54–1.43]) [29].

## 5. Limitations

Despite its promising efficacy and safety profile, this study has several limitations that should be acknowledged. First, the available evidence is largely based on a limited number of clinical trials with relatively small sample sizes conducted in geographically restricted populations. This may affect the generalizability of the results to broader groups, including individuals with significant comorbidities and diverse ethnic backgrounds.

Furthermore, the majority of the included studies assessed only short-term outcomes, providing little information about the long-term safety, effectiveness, and tolerability of etripamil. For example, the NODE-301 trial’s primary endpoint focused on termination of PSVT at 5 h, potentially underestimating the rapid action benefits of etripamil.

Future research should address these limitations by including larger and more diverse populations and conducting long-term follow-up studies to better assess persistent therapeutic outcomes and late adverse effects. Additionally, our meta-analysis was limited to data from only four randomized controlled trials, restricting the ability to perform more detailed subgroup analyses based on age, sex, comorbidities, or other important clinical factors.

As such, larger trials encompassing a wider range of populations and more detailed subgroup analyses are necessary to confirm and expand upon the findings of this review.

## 6. Conclusions

Etripamil exhibits significant promise as a future therapeutic option for the management of PSVT. Clinical trials have demonstrated its favorable effects in achieving conversion to a sinus rhythm and its relatively benign side effect profile compared with placebo and standard intravenous therapies. The ease of intranasal self-administration without the need for healthcare supervision enhances its potential utility in outpatient settings, low-resource areas, and among patients with limited access to immediate medical care.

However, to fully establish the role of etripamil in PSVT management, further studies with larger, more diverse patient populations are needed. Future research should also evaluate the safety and efficacy of etripamil in special populations, including pregnant women, children, and the elderly, and assess long-term outcomes following repeated or chronic use.

## Figures and Tables

**Table 1:** Study and patient characteristics.

Study	Country	Population Size	Male	Age (Years)	BMI (kg/m^2^)	Outcomes
Stambler et al., 2018 [20]	Canada USA	104	45 (43.3)	52.2 ± 13.13 ^a^	29.35 ± 8.98 ^a^	Etripamil nasal spray rapidly terminated induced SVT with a high conversion rate.
NODE-301 TRIAL [21]	Canada USA	196	67 (34.1)	56.8 ± 13.9 ^a^	29.2 ± 7.6	Etripamil self-administration during PSVT was safe and well tolerated. Although the primary 5 h efficacy endpoint was not met, the pooled analysis indicated an etripamil treatment effect in terminating PSVT.
RAPID TRIAL [22]	Belgium Canada France Germany Hungary Netherlands Poland Spain USA	184	53 (28.8)	54 ± 12	NR	Intranasal etripamil was well tolerated, safe, and superior to a placebo for the rapid conversion of atrioventricular nodal-dependent paroxysmal supraventricular tachycardia to a sinus rhythm.
Camm et al., 2023 [23]	Canada Netherlands	56	34 (60.7)	64.6 ± 10.47	NR	Intranasal etripamil (70 mg) reduced arrhythmic events and improved symptom relief and treatment satisfaction.

Values are reported as *n* (%) or mean ± standard deviation. ^a^ Mean age or standard deviation calculated from median and range. BMI = body mass index; NR = not reported.

## Data Availability

No new data sets were generated during this study.

## Supplementary Materials

The following supporting information can be downloaded at: https://www.mdpi.com/article/10.3390/jcm14113720/s1, Table S1: reporting the Preferred Reporting Items for Systematic Reviews and Meta-Analyses (PRISMA) checklist, Table S2: Table specifying reason for study exclusion, Figure S1: The PRISMA flow chart of the study; Figure S2: Sensitivity analysis for any adverse events (AEs), by excluding Camm et al., Figure S3: Risk of Bias Assessment Using the Cochrane Risk of Bias Tool (RoB-2) for Randomized Controlled Trials (RCTs).

## Abbreviations

PSVT	Paroxysmal supraventricular tachycardia
MeSH	Medical subject headings
PRISMA	Preferred Reporting Items for Systematic Reviews and Meta-Analyses
AV node	Atrioventricular node
AVNRT	Atrioventricular reentrant tachycardia
IV	Intravenous
BMI	Body mass index
NSVT	Non-sustained ventricular tachycardia
AEs	Adverse events
TESAEs	Treatment-emergent severe adverse events
AF	Atrial fibrillation
CCBs	Calcium channel blockers
