# Supplementary material for: Efficacy and Safety of Intranasal Etripamil for Paroxysmal Supraventricular Tachycardia: Meta-Analysis of Randomized Controlled Trials
Source: J Clin Med. 2025 May 26;14(11):3720. doi: 10.3390/jcm14113720 (PMC12155871; doi:10.3390/jcm14113720)

Table S1: Table reporting the Preferred Reporting Items for Systematic Reviews and Meta-Analyses (PRISMA) checklist.

Table S2: Table specifying reason for study exclusion.

Supplementary Figure S1: The PRISMA flow chart of the study.

Supplementary Figure S2: Sensitivity analysis for any adverse events (AEs), by excluding Camm et al.

Supplementary Figure S3: Risk of Bias Assessment Using the Cochrane Risk of Bias Tool (RoB-2) for Randomized Controlled Trials (RCTs).

Table S1: Table reporting the Preferred Reporting Items for Systematic Reviews and Meta-Analyses (PRISMA) checklist

PRISMA 2020 Checklist

Section and Topic	Item #	Checklist item	Location where item is reported
**TITLE**			
Title	1	Identify the report as a systematic review.	1
**ABSTRACT**			
Abstract	2	See the PRISMA 2020 for Abstracts checklist.	2
**INTRODUCTION**			
Rationale	3	Describe the rationale for the review in the context of existing knowledge.	5
Objectives	4	Provide an explicit statement of the objective(s) or question(s) the review addresses.	6
**METHODS**			
Eligibility criteria	5	Specify the inclusion and exclusion criteria for the review and how studies were grouped for the syntheses.	7
Information sources	6	Specify all databases, registers, websites, organisations, reference lists and other sources searched or consulted to identify studies. Specify the date when each source was last searched or consulted.	7
Search strategy	7	Present the full search strategies for all databases, registers and websites, including any filters and limits used.	7
Selection process	8	Specify the methods used to decide whether a study met the inclusion criteria of the review, including how many reviewers screened each record and each report retrieved, whether they worked independently, and if applicable, details of automation tools used in the process.	7-8
Data collection process	9	Specify the methods used to collect data from reports, including how many reviewers collected data from each report, whether they worked independently, any processes for obtaining or confirming data from study investigators, and if applicable, details of automation tools used in the process.	8
Data items	10a	List and define all outcomes for which data were sought. Specify whether all results that were compatible with each outcome domain in each study were sought (e.g. for all measures, time points, analyses), and if not, the methods used to decide which results to collect.	8
	10b	List and define all other variables for which data were sought (e.g. participant and intervention characteristics, funding sources). Describe any assumptions made about any missing or unclear information.	8-9
Study risk of bias assessment	11	Specify the methods used to assess risk of bias in the included studies, including details of the tool(s) used, how many reviewers assessed each study and whether they worked independently, and if applicable, details of automation tools used in the process.	8-9
Effect measures	12	Specify for each outcome the effect measure(s) (e.g. risk ratio, mean difference) used in the synthesis or presentation of results.	8-9
Synthesis methods	13a	Describe the processes used to decide which studies were eligible for each synthesis (e.g. tabulating the study intervention characteristics and comparing against the planned groups for each synthesis (item #5)).	8-9

	13b	Describe any methods required to prepare the data for presentation or synthesis, such as handling of missing summary statistics, or data conversions.	9
	13c	Describe any methods used to tabulate or visually display results of individual studies and syntheses.	9
	13d	Describe any methods used to synthesize results and provide a rationale for the choice(s). If meta-analysis was performed, describe the model(s), method(s) to identify the presence and extent of statistical heterogeneity, and software package(s) used.	9
	13e	Describe any methods used to explore possible causes of heterogeneity among study results (e.g. subgroup analysis, meta-regression).	9
	13f	Describe any sensitivity analyses conducted to assess robustness of the synthesized results.	9
Reporting bias assessment	14	Describe any methods used to assess risk of bias due to missing results in a synthesis (arising from reporting biases).	9

PRISMA 2020 Checklist

Section and Topic	Item #	Checklist item	Location where item is reported
Certainty assessment	15	Describe any methods used to assess certainty (or confidence) in the body of evidence for an outcome.	9
**RESULTS**			
Study selection	16a	Describe the results of the search and selection process, from the number of records identified in the search to the number of studies included in the review, ideally using a flow diagram.	9
	16b	Cite studies that might appear to meet the inclusion criteria, but which were excluded, and explain why they were excluded.	10
Study characteristics	17	Cite each included study and present its characteristics.	10
Risk of bias in studies	18	Present assessments of risk of bias for each included study.	12
Results of individual studies	19	For all outcomes, present, for each study: (a) summary statistics for each group (where appropriate) and (b) an effect estimate and its precision (e.g. confidence/credible interval), ideally using structured tables or plots.	10-11
Results of syntheses	20a	For each synthesis, briefly summarise the characteristics and risk of bias among contributing studies.	11
	20b	Present results of all statistical syntheses conducted. If meta-analysis was done, present for each the summary estimate and its precision (e.g. confidence/credible interval) and measures of statistical heterogeneity. If comparing groups, describe the direction of the effect.	11
	20c	Present results of all investigations of possible causes of heterogeneity among study results.	11-12
	20d	Present results of all sensitivity analyses conducted to assess the robustness of the synthesized results.	11-12
Reporting biases	21	Present assessments of risk of bias due to missing results (arising from reporting biases) for each synthesis assessed.	12

Certainty of evidence	22	Present assessments of certainty (or confidence) in the body of evidence for each outcome assessed.	12
**DISCUSSION**			
Discussion	23a	Provide a general interpretation of the results in the context of other evidence.	12-13
	23b	Discuss any limitations of the evidence included in the review.	13-14
	23c	Discuss any limitations of the review processes used.	14-15
	23d	Discuss implications of the results for practice, policy, and future research.	15-16
**OTHER INFORMATION**			
Registration and protocol	24a	Provide registration information for the review, including register name and registration number, or state that the review was not registered.	7
	24b	Indicate where the review protocol can be accessed, or state that a protocol was not prepared.	7
	24c	Describe and explain any amendments to information provided at registration or in the protocol.	7
Support	25	Describe sources of financial or non-financial support for the review, and the role of the funders or sponsors in the review.	17
Competing interests	26	Declare any competing interests of review authors.	17
Availability of data, code and other materials	27	Report which of the following are publicly available and where they can be found: template data collection forms; data extracted from included studies; data used for all analyses; analytic code; any other materials used in the review.	17

PRISMA 2020 Checklist

From: Page MJ, McKenzie JE, Bossuyt PM, Boutron I, Hoffmann TC, Mulrow CD, et al. The PRISMA 2020 statement: an updated guideline for reporting systematic reviews. BMJ 2021;372:n71. doi: 10.1136/bmj.n71

For more information, visit: http://www.prisma-statement.org/

Table S2: Excluded Studies and Reasons for Exclusion

No.	First Author (Year)	Title (Shortened)	Reason for Exclusion
1	Pokorney SD (2025)	Secondary analysis of RCT (ED visits)	Secondary analysis, not a primary RCT
2	Ascah A (2025)	Intravenous etripamil in telemetered monkeys	Preclinical animal study
3	Roberts L (2025)	Self-administered intranasal etripamil: a new treatment to keep SVT out of the ED?	Commentary/editorial
4	Stambler BS (2024)	Symptom-prompted open-label intranasal etripamil for AVN-dependent SVT	Non-randomized, open-label study
5	Macech A (2024)	Network meta-analysis of etripamil in acute PSVT conversion	Meta-analysis, not original clinical trial
6	Pion J (2024)	Preclinical safety evaluation in cynomolgus macaques	Animal toxicology study
7	Calvert P (2024)	Intranasal etripamil for rapid PSVT treatment (review)	Review article, not original research
8	Ip JE (2024)	Etripamil nasal spray for recurrent PSVT conversion	Single-arm or case series, not an RCT

9	Ip JE (2024)	Plain-language summary of RAPID safety/effectiveness	Summary paper, not a standalone RCT
10	Ip JE (2024)	Pharmacokinetics & pharmacodynamics of intranasal etripamil	Phase 1 PK/PD study, not clinical efficacy
11	Ip JE (2024)	Rationale and design of NODE-303 (protocol only)	Study protocol without results
12	Papakyriakopoulou P (2024)	"Nose-to-Heart" review of intranasal drug delivery	Review article
13	Strzelczyk E (2024)	Etripamil for AVN-dependent SVT ([German])	Non-English report, no efficacy data
14	Stambler BS (2023)	Podcast on intranasal etripamil for PSVT (RAPID)	Podcast commentary, not original research
15	Ip JE (2023)	NODE-302 open-label extension for repeated PSVT episodes	Open-label extension, not randomized
16	Huston J (2023)	Correction to therapeutic potential review	Erratum, not original data
17	Huynh K (2023)	Rapid return to sinus rhythm after SVT using etripamil (brief report)	Brief report, not RCT
18	Abuelazm M (2023)	Systematic review & meta-analysis of RCTs in PSVT	Secondary synthesis, not primary trial
19	Huston J (2023)	Therapeutic potential review of etripamil	Review article

20	Stambler BS (2022)	Design of phase 3, placebo-controlled etripamil study (protocol)	Protocol/design only
21	Chu GS (2021)	Update on at-home treatment of acute PSVT with etripamil	Review/update article
22	Weintraub S (2021)	Future of intranasal drug delivery for arrhythmias	Review article
23	Kashou AH (2020)	Investigational agent for rapid SVT termination	Review article
24	Raja JM (2021)	Intranasal calcium-channel blocker: novel modality for PSVT	Review article
25	Faisaluddin M (2019)	Self-management of SVT: intranasal etripamil commentary	Commentary, not original trial
26	Choe WC (2017)	High-density mapping after intranasal etripamil during NODE-1	Case report, not randomized trial

Supplementary Figure S1: The PRISMA flow chart of the study.

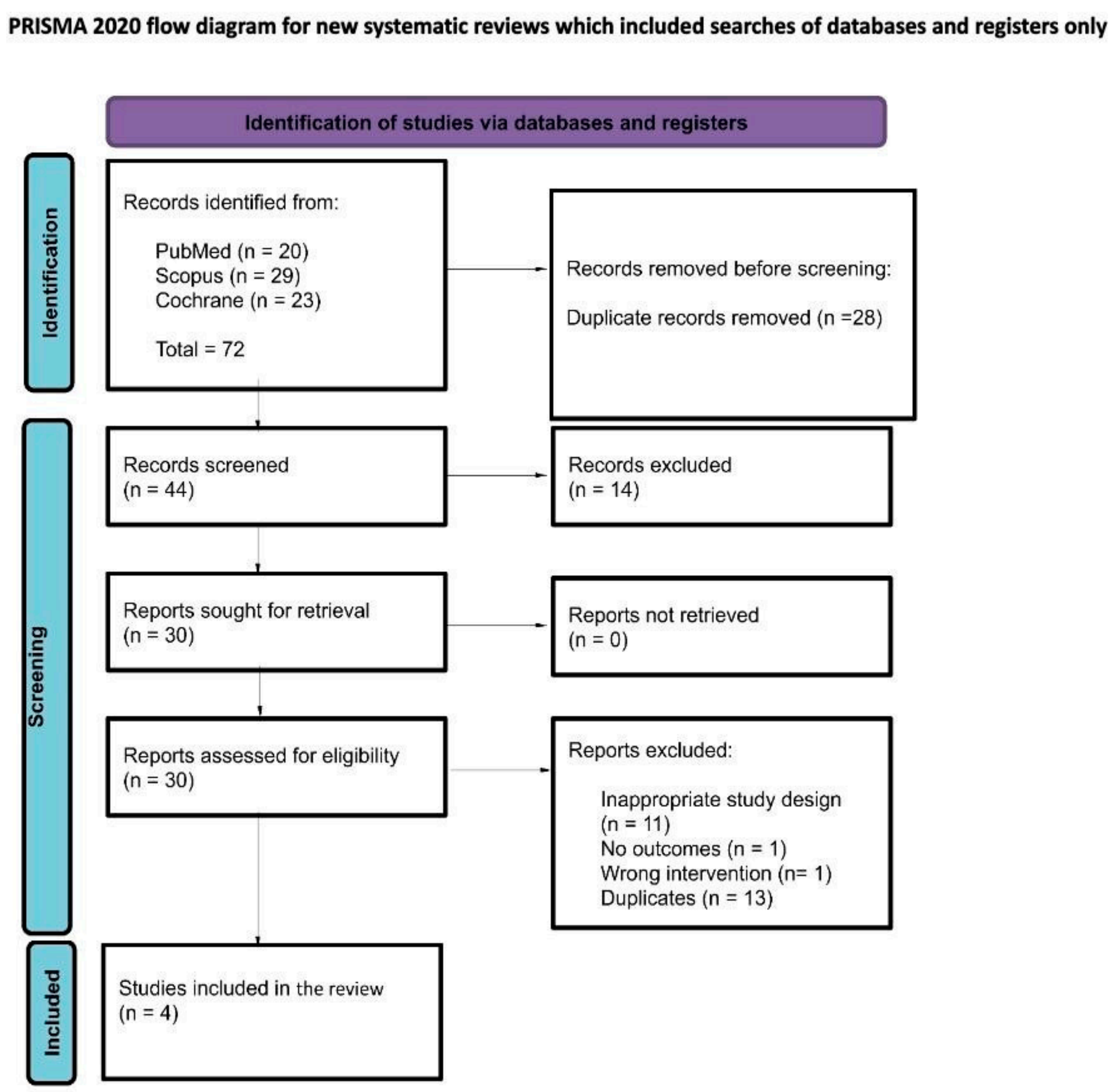

From: Page MJ, McKenzie JE, Bossuyt PM, Boutron I, Hoffmann TC, Mulrow CD, et al. The PRISMA 2020 statement: an updated guideline for reporting systematic reviews. BMJ 2021;372:n71. doi: 10.1136/bmj.n71

For more information, visit: http://www.prisma-statement.org/

Supplementary Figure S2: Sensitivity analysis for any adverse events (AEs), by excluding Camm et al.

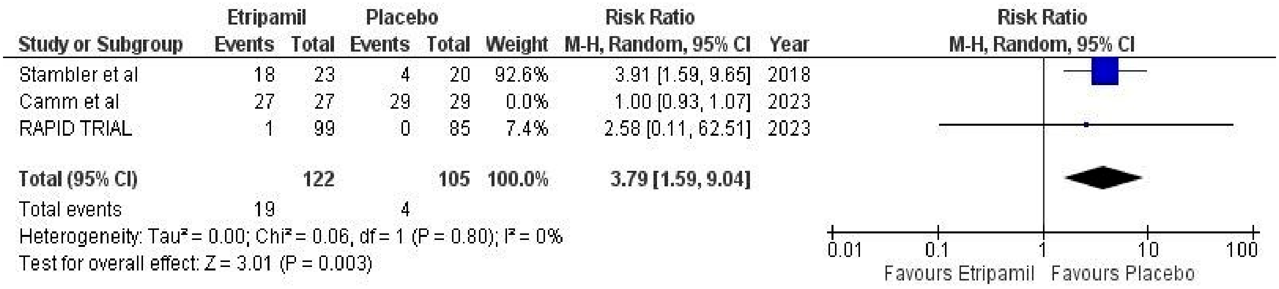

Supplementary Figure S3: Risk of Bias Assessment Using the Cochrane Risk of Bias Tool (RoB-2) for Randomized Controlled Trials (RCTs).

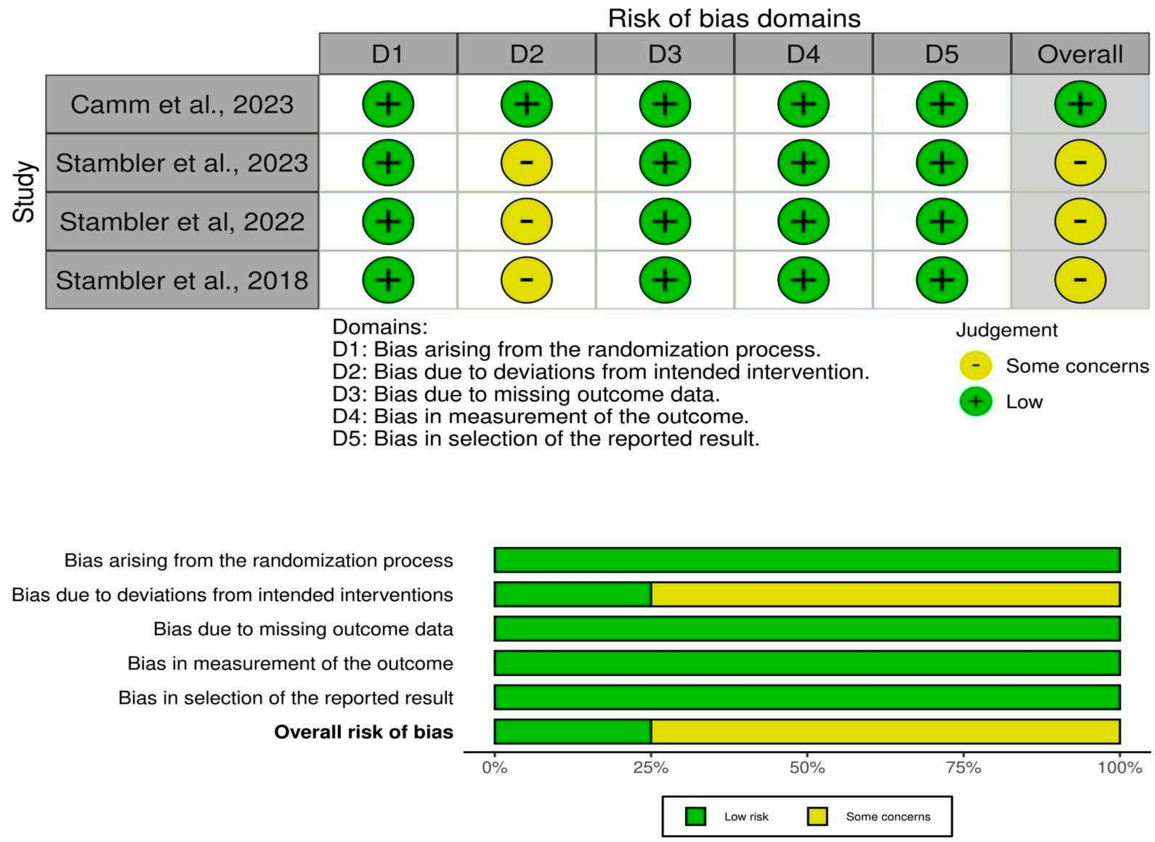

Supplement: Supplementary file 1 [file jcm-14-03720-s001.zip › jcm-3608025-supplementary.pdf]
